# Effects of crocin on spatial or aversive learning and memory impairments induced by lipopolysaccharide in rats 

**Published:** 2021

**Authors:** Mohammad Javad Azmand, Ziba Rajaei

**Affiliations:** *Department of Physiology, School of Medicine, Isfahan University of Medical Sciences, Isfahan, Iran*

**Keywords:** Crocin, Lipopolysaccharide, Memory, Interleukin-1β, Oxidative stress, Systemic inflammation

## Abstract

**Objective:**

Neuroinflammation and oxidative stress play essential roles in the pathogenesis and progression of neurodegenerative diseases, such as Alzheimer’s disease. Crocin, main active constituent of *Crocus sativus* L. (saffron), possesses anti-inflammatory, anti-apoptotic and anti-oxidative capacity. The aim of the present study was to investigate the neuroprotective effect of crocin on lipopolysaccharide (LPS)-induced learning and memory deficits and neuroinflammation in rats.

**Materials and Methods:**

The animals were randomly classified into four groups, including control, LPS, crocin 50 and crocin 100. The rats were treated with either crocin (50 and 100 mg/kg) or saline for a week. Later, LPS (1 mg/kg, i.p.) or saline was administered, and treatments with crocin or saline were continued for 3 more weeks. The behavioral tasks for spatial and aversive memories were performed by the Morris water maze and passive avoidance tasks from post-injection days 18 to 24. Furthermore, the levels of interleukine-1β, lipid peroxidation and total thiol were assayed in the hippocampus and cerebral cortex.

**Results:**

Our results demonstrated that treatment of LPS-treated rats with crocin decreased the escape latency in the Morris water maze and increased the time spent in the target quadrant in the probe trial. Moreover, crocin increased step-through latency in the passive avoidance test. However, there was no significant difference in the oxidative and neuroinflammatory responses among the experimental groups.

**Conclusion:**

Pretreatment with crocin attenuates spatial or aversive learning and memory deficits in LPS-treated rats.

## Introduction

Neuroinflammation is considered as a crucial mechanism in the pathogenesis and progression of neurodegenerative diseases, particularly Alzheimer's disease (Sandra et al., 2010). Neuroinflammation is characterized by overactivation of neuroglia and overproduction of proinflammatory mediators. Once overactivated, microglia release various proinflammatory cytokines, including tumor necrosis factor-α (TNF-α), interleukin-1β (IL-1β) and interleukin-6 (IL-6) in the CNS, which induce detrimental effects on neurons (Yirmiya and Goshen, 2011; Tseng et al., 2012). Neuronal death in the brain regions, such as cerebral cortex and hippocampus, impairs learning and memory and other cognitive functions (Dinel et al., 2011; Chang et al., 2013). Experimental data demonstrates that neuroinflammation results in spatial memory impairment (Hauss-Wegryzniak et al., 1998).

Lipopolysaccharide (LPS) is a potent bacterial endotoxin which induces microglia activation and increases secretion of proinflammatory cytokines, including IL-1β and TNF­α (Qin et al., 2007; Henry et al., 2009). LPS is commonly applied to induce animal models of neuroinflammation for investigating the association between inflammation and memory deficits (Zhan et al., 2018; Thomson and Sutherland, 2005). It has been reported that systemic administration of LPS induces neuroinflammation in the frontal cortex and hippocampus (Noh et al., 2014), causes neuronal death (Cunningham et al., 2005) and impairs learning and memory in animal models (Lee et al., 2008; Gholami et al., 2019). 

It has also been demonstrated that microglia and macrophages activated by LPS, produces oxygen and nitrogen free radicals, leading to the neurodegenerative processes (Wang et al., 2004; Qin et al., 2007). Oxidative stress induced by free radicals damages neuronal membrane components (lipids, proteins, and DNA), leading to neuronal dysfunction (Lobo et al., 2010; Moreira et al., 2008).

Recent evidence implies that improvement or prevention of the detrimental effects of inflammation and oxidative damage is beneficial in preventing Alzheimer's disease onset and retarding cognitive dysfunction (Daultzer, 2016).

Crocin is a glycosylated carotenoid and the main constituent of the stigma of saffron (*Crocus sativus *L.). Crocin possesses anti-apoptotic (Qi et al., 2013), anti-inflammatory (Nam et al., 2010) and antioxidant (Chen et al., 2008; Rajaei et al., 2013) activities. Crocin was shown to inhibit microglial activation and decrease the expression of proinflammatory mediators (IL-1β, TNF-α, inducible nitric oxide synthase (iNOS), cyclooxygenase-2 (COX2)) induced by LPS *in vitro *(Nam et al. 2010; Lv et al. 2016). Further, crocin decreased LPS-stimulated reactive oxygen species release from microglial cells of rat brain (Nam et al. 2010). It has also been shown that crocin improves learning and memory deficits in diabetic rats by decreasing oxidative damage in the cerebral cortex (Ahmadi et al., 2017). 

The current study was designed to examine the effect of crocin pretreatment on systemic LPS-induced neuroinflammation and memory impairments in a rat model. The levels of IL-1β and oxidative stress markers were measured in the hippocampus and cerebral cortex to find the potential mechanism (s) of action of crocin. 

## Materials and Methods

**Animals**

Male Wistar rats (220-250 g) procured from Royan Institute (Isfahan, Iran), were housed in an air-conditioned room at 22±2°C with a 12hr light/dark cycle and they had free access to food and water. The Ethic Committee for Animal Experiments at Isfahan University of Medical Sciences approved the study (IR.MUI.REC.1396.1.067) and all experiments were conducted in accordance with the National Institute of Health Guide for the Care and Use of Laboratory Animals (NIH Publication, 8th edition, 2011).

**Treatment schedule**

Animals were randomly divided into four experimental groups (n=10 in each group), including: 1. Control group which was treated with normal saline for 4 weeks. 

2. LPS group which was treated with normal saline for 4 weeks. LPS (1 mg/kg) (Vasconcelos et al., 2014) was also administered intraperitoneally (i.p.) to this group on day 0. 

3. Crocin 50 group which was treated with crocin (50 mg/kg, i.p.) for 4 weeks and LPS (1 mg/kg, i.p.) was administered on day 0. 

4. Crocin 100 group which was treated with crocin (100 mg/kg, i.p.) for 4 weeks. LPS (1 mg/kg, i.p.) was administered on day 0. 

**Figure 1:**
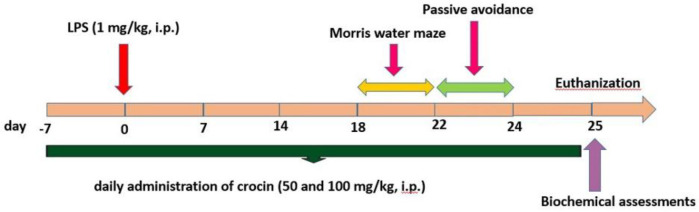
Timeline representing treatment of crocin and LPS, as well as assessment of behavioral and biochemical parameters in rats

Crocin (Sigma-Aldrich Co., USA) was freshly prepared in normal saline prior to injection. LPS from *Escherichia coli* (Sigma-Aldrich Co., USA) was freshly dissolved in normal saline. Injection of crocin or normal saline started one week before LPS injection and continued for 3 more weeks after LPS injection. The schedule of the study was determined according to a previous study (Hou et al., 2014) as well as our preliminary experiments. Two weeks after LPS administration, the behavioral tests (Morris water maze and passive avoidance test) were done. The outline of the treatment schedule, and behavioral and biochemical assessments is shown in Figure 1. 

**Behavioral assessments**

**Morris water maze**

Spatial learning and memory was evaluated by the Morris water maze task on days 18-22 after LPS injection. The black circular pool (150 cm in diameter and 50 cm height) was filled with tap water (24±1°C) and surrounded by various cues for spatial orientation. The pool was divided into four quadrants, and four releasing positions were designed as follows: North-east, North-west, South-east and South-west. In the spatial acquisition phase, the rats learned to find a submerged platform using extra-maze cues. An escape circular platform (10 cm in diameter) was submerged 2 cm below the surface of water in south-east quadrant of the pool. In the spatial acquisition phase, the rats performed four 60-sec trials on each of the four consecutive days. In all trials, rats had to swim, until they find the platform and escape from the water. If a rat could not find the platform within 60 sec, it was directed to the platform and stayed on it for 30 sec. A computer software (NeuroVision, TajhizGostar Co.) was used to calculate the escape latency and traveled distance to find the platform, for each rat. A 60-sec probe test was performed on day 22 with the platform removed from the pool. A computer software was used to calculate the time spent in the south-east quadrant (Ahmadi et al., 2017). 

**Passive avoidance memory**

Passive avoidance apparatus is made of light and dark chambers, connected by a guillotine door. The test was performed on days 22-24 at the same time of the day. During habituation on day 22, rats were placed into the apparatus for 5 min to move freely between the two chambers. In acquisition phase that was conducted 24 hr later, rats were located in the light chamber. Upon entry of the rat to the dark chamber, a 0.8 mA electric shock was delivered to the feet for 3 sec. In retention phase that was performed on day 24, the rats were again placed into the light chamber and the step-through latency to enter the dark chamber, was measured (Shahidani et al., 2019). 

**Tissue collection and homogenization**

After behavioral assessments, animals were euthanized and their brains were removed. Then, the hippocampus and cerebral cortex (approximately 400 mg) were dissected and weighed. A 10% (w/v) tissue homogenate was prepared for biochemical assessments. All chemicals for biochemical measurements (TBARS and total thiol concentration) were purchased from Merck Co. (Germany).

**Biochemical assessments**

**IL-1β levels**

Cortical and hippocampal homogenates were centrifuged at 3000 rpm for 5 min. Later, the IL-1β levels in the supernatants were determined using specific ELISA kits (BMS 630, ebioscience) and according to the manufacturer's instructions. Results are shown as pg/ml.

**Lipid peroxidation levels**

Lipid peroxidation levels of the hippocampus and cortex were measured as TBARS (thiobarbituric acid reactive substances), which is the end-product of lipid peroxidation. For determination of TBARS levels, trichloroacetic acid, thiobarbituric acid and HCl were mixed and added to the homogenate. Then, the mixture was incubated in boiling water for 45 min. After cooling, the samples were centrifuged at 1000 g for 10 min and the absorbance of the supernatant was read at 535 nm. The TBARS levels was calculated by: C(M) = Absorbance/1.65 × 10^5 ^(Rajaei et al., 2013). 

**Total thiol concentration**

Total sulfhydryl groups level was measured in the hippocampus and cortex using DTNB (2,2´-dinitro-5,5´-dithiodibenzoic acid) as the reagent. For this purpose, tissue homogenate was added to tris-EDTA buffer and the absorbance was read at 412 nm (A1). Later, the DTNB reagent was added to the samples. After 15 min, the absorbance was read again (A2). The absorbance of the DTNB reagent was read as a blank (B). The total thiol concentration (mM) was calculated by: (A2-A1-B)×1.07/0.05×13.6 (Rajaei et al., 2013). 

**Statistical analysis**

Data are expressed as mean±SEM. Statistical analysis was carried out using one-way ANOVA (for probe trial, passive avoidance and biochemical data) and two-way repeated measures ANOVA (for acquisition training over 4 days) followed by Tukey’s *post hoc* test. A p<0.05 was considered significant.

## Results

**Effects of crocin on spatial learning and memory deficits**

**Figure 2:**
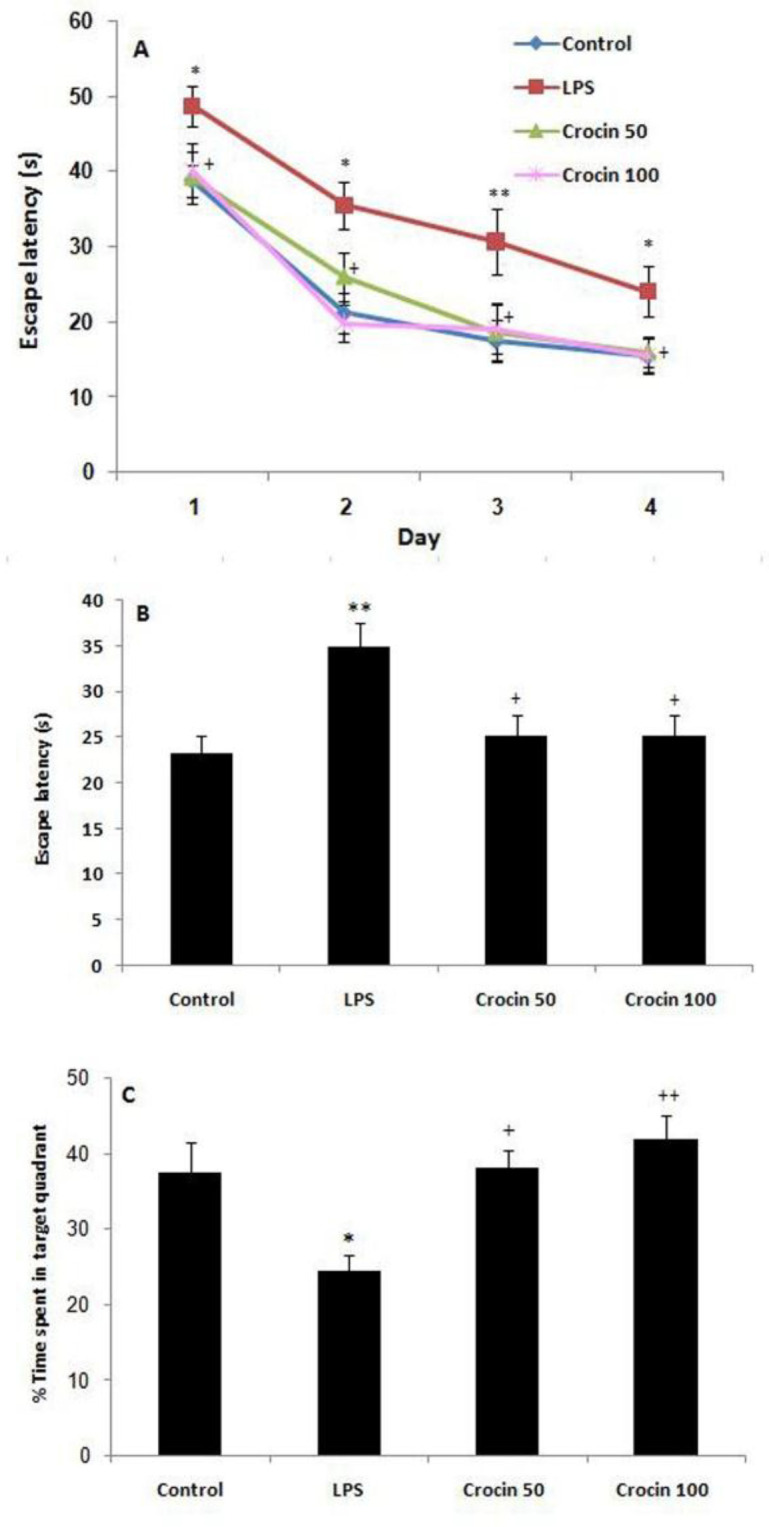
Effects of crocin on the performance of spatial memory acquisition phase in Morris water maze, (A) Escape latency during 4 days, (B) overall escape latency, (C) performance in probe trial. Data are shown as mean±SEM for ten animals in each group. ^*^p<0.05 and ^**^p<0.01 *vs* control group and ^+^p<0.05 and ^++^p<0.01 *vs* LPS group

Statistical analysis using two-way repeated measures ANOVA revealed that the escape latency to find the platform decreased over four learning days in all groups, indicating spatial learning acquisition (F_(3,81)_=51.12, p<0.001, Figure 2A). Moreover, the LPS-treated rats showed higher escape latency (F_(3,36)_=5.73, p<0.01, Figures 2A and 2B) compared to the control rats. This indicates that LPS injection impaired the spatial learning acquisition phase in Morris water maze. Furthermore, treatment of rats with crocin at doses of 50 and 100 mg/kg, significantly decreased escape latency (p<0.01, Figures 2A and 2B) compared to the LPS rats. 

In the probe trial performed on day 22, LPS group spent less time in the target quadrant compared to the control group (F_(3,36)_=6.41, p<0.05, Figure 2C). Moreover, crocin 50 and crocin 100 groups spent more time in the target quadrant compared to the LPS group (p<0.05, p<0.01, respectively; Figure 2C).

**Effects of crocin on p** **assive avoidance memory**

**Figure 3:**
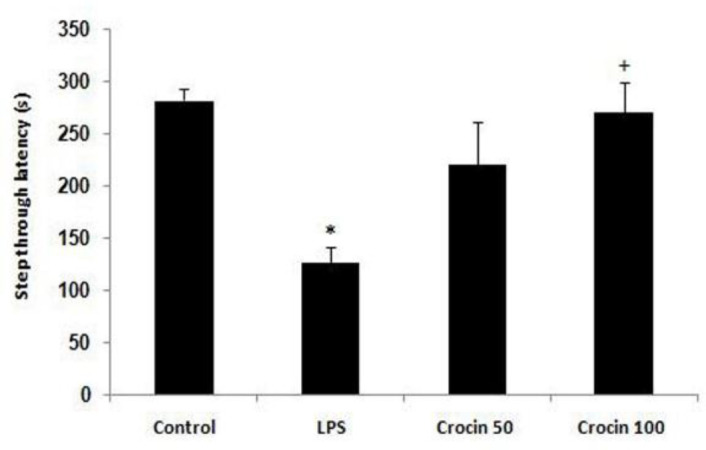
Step-through latency in the control and LPS-treated rats. Data are shown as mean±SEM for ten animals in each group. *p<0.05 *vs* control group, and ^+^p<0.05 *vs* LPS group

As shown in Figure 3, the step-through latency of LPS-treated rats was shorter than control group rats at the end of the experiment (p<0.05, Figure 3). Further, treatment with crocin at a dose of 100 mg/kg significantly increased the latency compared to LPS-treated group (p<0.05). 

**Effects of crocin on IL-1β levels**

**Figure 4:**
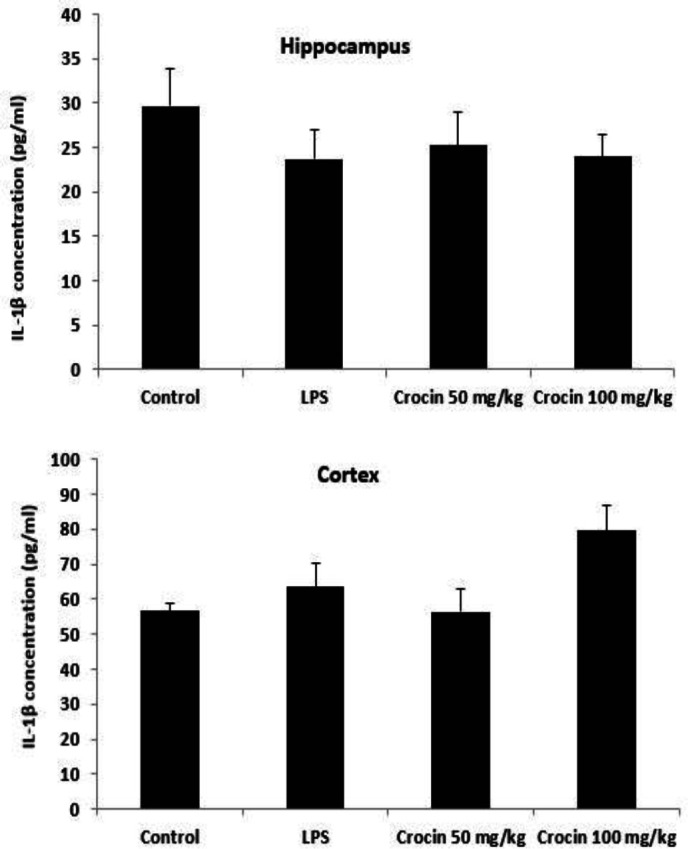
Effects of crocin on IL-1ß levels in the hippocampus and cerebral cortex of control and LPS-treated rats. Data are shown as mean±SEM for ten animals in each group

Data analysis revealed that there was no significant difference in IL-1β levels in the hippocampus (F_(3,36)_=0.6, p>0.05, Figure 4) and cortex between control and LPS-treated groups (F_(3,36)_=3.16, p>0.05, Figure 4).

**Effects of crocin on lipid peroxidation levels**

**Figure 5:**
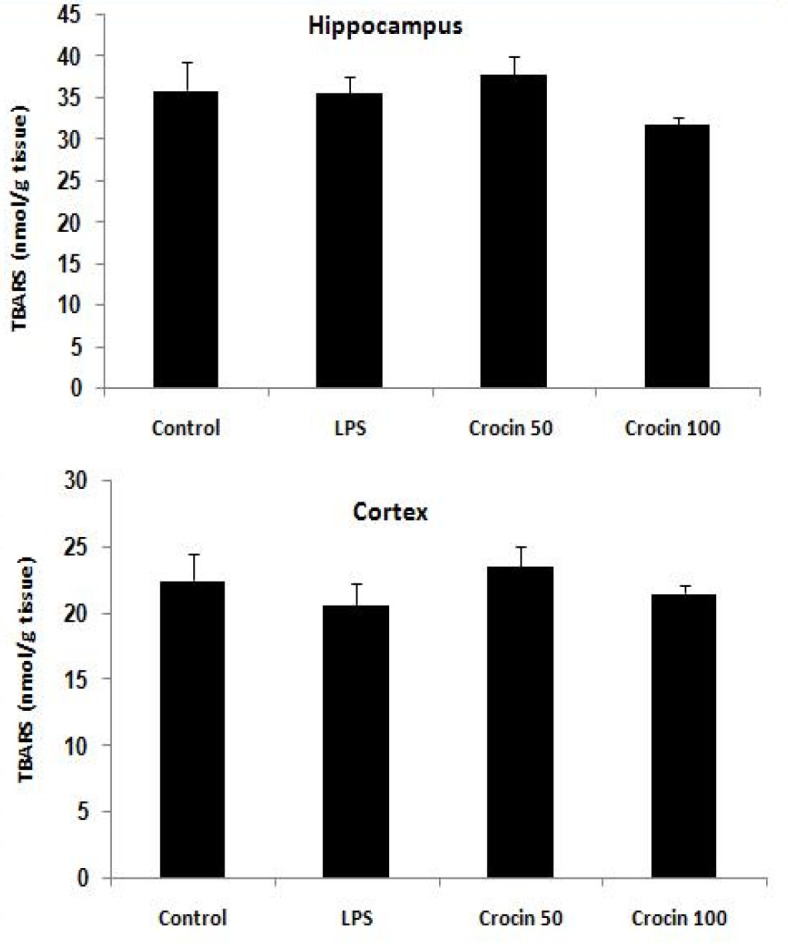
Effects of crocin on lipid peroxidation levels (TBARS) in the hippocampus and cerebral cortex of control and LPS-treated rats. Data are shown as mean±SEM for ten animals in each group

As Figure 5 shows, there was no significant change in TBARS levels, as an index of lipid peroxidation, in the hippocampus (F_(3,36)_=1.26, p>0.05) and cortex (F_(3,36)_=0.67, p>0.05)between control and LPS-treated groups at the end of the experiment. 

**Figure 6.**
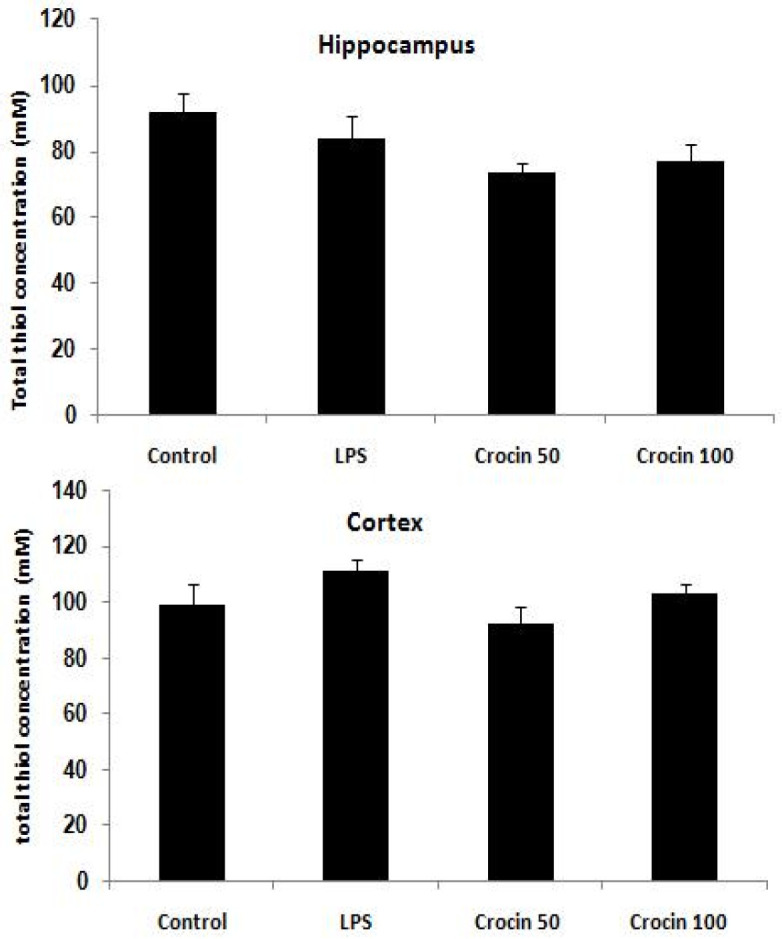
Effects of crocin on total thiol concentration in the hippocampus and cerebral cortex of control and LPS-treated rats. Data are shown as mean±SEM for ten animals in each group

**Effects of crocin on total thiol concentration**

Figure 6 shows total thiol concentration in the hippocampus and cortex of the control and experimental groups. There was no significant difference in total thiol concentration in the hippocampus (F_(3,36)_=2.74, p>0.05) and cortex (F_(3,36)_=2.03, p>0.05) between control and LPS-treated groups at the end of the experiment. 

## Discussion

Our results indicated that LPS administration caused spatial and aversive memory deficits in rats. Moreover, pretreatment with crocin at doses of 50 and 100 mg/kg, improved learning and memory deficits induced by LPS. 

LPS-induced memory impairment has been used as an experimental approach for identifying the mechanisms involved in the pathophysiology of Alzheimer’s disease (Czerniawski et al., 2015). In this study, we evaluated memory impairment using Morris water maze which represents a spatial memory task (Götz and Ittner, 2008). We observed that systemic injection of LPS impaired spatial learning and memory, since LPS-treated animals showed an increase in the escape latency to find the platform and a significant decrease in time spent in the target quadrant in the probe trial. We also used passive avoidance task to evaluate aversive memory in LPS-treated rats. In this task, animals learn to keep away from an environment in which an aversive stimulus (such as a foot-shock) was already delivered. Our data indicated that LPS administration significantly decreased step-through latency in treated rats. These results are in agreement with previous studies which have demonstrated that systemic injection of LPS impairs spatial or aversive learning and memory (Shaw et al., 2001; Lee et al., 2008; Gholami et al., 2019). In our study, crocin ameliorated the spatial and aversive memory deficits in rats, because it decreased the escape latency time in the Morris water maze and increased the time spent in the target quadrant in the probe trial. Moreover, crocin increased step-through latency in the shuttle box. In agreement with our results, previous reports have also indicated the beneficial effects of crocin on memory deficits in several experimental models. For example, it was shown that crocin attenuated spatial learning and memory impairments in diabetic rats (Ahmadi et al., 2017). Furthermore, crocin could improve retention in passive avoidance task in a 6-hydroxydopamine (6-OHDA) model of Parkinson’s disease (Rajaei et al., 2016). 

Experimental evidence indicated that administration of LPS results in memory impairments through mechanisms that affect expression of pro-inflammatory cytokines (Bossu et al., 2012; Lee et al., 2018), oxidative stress (Ammari et al., 2018), cholinergic dysfunction and neuronal death (Semmler et al., 2007). 

LPS enhances the synthesis and release of proinflammatory mediators, which initiate a series of pathological events in the periphery and the CNS, eventually leading to dysfunctional memory consolidation and memory decline (Daulatzai, 2016). Proinflammatory cytokines, such as TNF-α and IL-1β are involved in hippocampal long-term potentiation and dendritic arborization, which are key processes underlying the formation and long-term storage of memories (Kim and Diamond, 2002). However, activation of microglia by LPS and increased proinflammatory cytokines, results in synaptic plasticity impairments, neurogenesis failure, neuronal cell death and memory deficits (Monje et al., 2003; Cunningham 2005; Lee et al., 2008). In support of this, it was shown that administration of LPS to Wistar rats increased the expression of TNF-α, IL-1β, and IL-6 in the hippocampus (Daulatzai, 2016). Beta amyloid level was also increased in the hippocampus following seven days of LPS administration, which was accompanied by memory dysfunction in Morris maze test (Shaw et al., 2001; Zhu et al., 2014). It has been demonstrated that the inflammatory reaction that develops in the hippocampus and temporal lobe after chronic LPS administration, lasts for a long time and underlie memory deficits in the Morris water maze task (Hauss-Wegrzyniak et al., 2000).

Evidence indicates that crocin exhibits anti-inflammatory activity (Nam et al., 2010; Lv et al., 2016). For instance, it has been shown that crocin decreases LPS-induced production of TNF­α, and IL-1β byactivated microglia (Nam et al., 2010). In another study, crocin was shown to suppress LPS-induced microglial activation and decrease the expression of pro-inflammatory mediators, such as IL-1β and TNF-α (Lv et al., 2016). Further, anti-inflammatory effects of crocin by decreasing TNF-α, have been shown in hemorrhagic shock (Yang and Dong, 2017) and rheumatoid arthritis (Li et al., 2017). Accordingly, the positive effect of crocin on LPS-induced memory deficits in the current study could be partly due to its anti-inflammatory activity.

However, in the present study, there was no significant difference in IL-1ß levels in the hippocampus and cortex between control and LPS groups. The reason might be due to measurement of IL-1β level 3 weeks after LPS injection, when memory deficits were observed. Other studies have shown that LPS increased TNF-α and IL-1β levels in the hippocampus 6 hr after the injection, while cytokine levels returned to the baseline levels 24 hr post-injection (Zhang et al., 2015; Terrando et al., 2010). Therefore, crocin might have exerted its anti-inflammatory activity early during LPS injection. 

Brain tissue oxidative damage also has been regarded as a significant mechanism for LPS-induced memory deficits (Hritcu et al., 2011). Brain is more susceptible to oxidative stress because it has high concentrations of polyunsaturated fatty acids (Montine et al., 2002), high oxygen demand and low levels of antioxidants (Moreira et al., 2008). It has been reported that oxidative damage to the hippocampal and cortical synapses contribute to memory impairments (Ammari et al., 2018; Ahmadi et al., 2017). Evidence has demonstrated that crocin possesses free radical scavenging (Assimopoulou et al., 2005) and antioxidant activities, inhibits the formation of peroxidized lipids and restores superoxide dismutase activity (Ochiai et al., 2004). It has also been reported that crocin improves spatial memory deficits in diabetic rats by reducing oxidative damage in the cerebral cortex. Moreover, crocin decreases lipid peroxidation and nitrite levels in the hippocampus, and improves aversive memory in a 6-OHDA model of Parkinson’s disease (Rajaei et al., 2016). Therefore, the beneficial effect of crocin on memory deficits induced by LPS could also be partly due to its antioxidant activity. 

In the current study, there was no significant change in oxidative stress markers levels in rat brain after LPS injection. This might be due to measurement of oxidative stress biomarkers 3 weeks after LPS injection. In support of this, Theobaldo et al. (2012) have reported that malondialdehyde levels highly increased 1.5 hr after LPS injection, while it approached baseline levels 24 hr post-LPS (Theobaldo et al., 2012). Collectively, it could be concluded that the improvement of memory deficits by crocin could be partly due to its antioxidant activity against early LPS oxidative damage.

Several studies have shown a correlation between memory deficits and cholinergic dysfunction following LPS injection (Tyagi et al., 2010). It has been reported that systemic LPS impairs memory function via increase in acetylcholinesterase activity (Tyagi et al., 2010) and reduction of acetylcholine (Houdek et al., 2014; Willard et al., 1999). It has also been shown that the crocin attenuates scopolamine-induced memory impairments in rats (Pitsikas et al., 2007). It has been reported that saffron is a source of acetylcholinesterase inhibitors (Geromichalos et al., 2012) and crocin may compose more than 10% of dry saffron's mass (Abdullaev, 2002). Thus, the impact of crocin on improvement of memory in LPS-treated rats could also be attributed to its anticholinesterase activity, although this mechanism was not evaluated in the current study.

It should be noted that LPS could induce memory deficits by other mechanisms, such as inhibition of hippocampal synaptic plasticity (Hennigan et al., 2007), decreasing hippocampal BDNF mRNA levels (Kranjac et al., 2012; Amraie et al., 2020), impairment of hippocampal neurogenesis (Valero et al., 2014), enhancement of beta-amyloid formation (Lee et al., 2008; Ali et al., 2016), and apoptosis (Wang et al., 2011; Zarifkar et al., 2010). Previous studies have indicated that crocin potentiates synaptic plasticity (Abe and saito, 2000), increases hippocampal BDNF levels (Behravanfar et al., 2017; Mozaffari et al., 2019), inhibits beta-amyloid generation (Ghahghaei et al., 2013) and prevents apoptosis (Asadi et al., 2015; Mohammadzadeh et al., 2019). Therefore, above mechanisms may also play roles in crocin ameliorative effect on memory deficits. 

In conclusion, the present study confirmed that pre-treatment with crocin improves spatial or aversive learning and memory deficits in LPS-treated rats. To the best of our knowledge, this is the first report that provided evidence about beneficial effects of crocin on memory deficits in LPS-treated rats. 
